# Malondialdehyde Acetaldehyde-Adduction Changes Surfactant Protein D Structure and Function

**DOI:** 10.3389/fimmu.2022.866795

**Published:** 2022-05-20

**Authors:** Claire G. Nissen, Deanna D. Mosley, Kusum K. Kharbanda, Dawn M. Katafiasz, Kristina L. Bailey, Todd A. Wyatt

**Affiliations:** ^1^Department of Environmental, Agricultural and Occupational Health, College of Public Health, University of Nebraska Medical Center, Omaha, NE, United States; ^2^Department of Internal Medicine, College of Medicine, University of Nebraska Medical Center, Omaha, NE, United States; ^3^Research Service Veterans Affairs Nebraska-Western Iowa Health Care System, Omaha, NE, United States

**Keywords:** alcohol, lung, pneumonia, surfactant, aldehydes, adduction

## Abstract

Alcohol consumption with concurrent cigarette smoking produces malondialdehyde acetaldehyde (MAA)-adducted lung proteins. Lung surfactant protein D (SPD) supports innate immunity *via* bacterial aggregation and lysis, as well as by enhancing macrophage-binding and phagocytosis. MAA-adducted SPD (SPD-MAA) has negative effects on lung cilia beating, macrophage function, and epithelial cell injury repair. Because changes in SPD multimer structure are known to impact SPD function, we hypothesized that MAA-adduction changes both SPD structure and function. Purified human SPD and SPD-MAA (1 mg/mL) were resolved by gel filtration using Sephadex G-200 and protein concentration of each fraction determined by Bradford assay. Fractions were immobilized onto nitrocellulose by slot blot and assayed by Western blot using antibodies to SPD and to MAA. Binding of SPD and SPD-MAA was determined fluorometrically using GFP-labeled *Streptococcus pneumoniae* (GFP-SP). Anti-bacterial aggregation of GFP-SP and macrophage bacterial phagocytosis were assayed by microscopy and permeability determined by bacterial phosphatase release. Viral injury was measured as LDH release in RSV-treated airway epithelial cells. Three sizes of SPD were resolved by gel chromatography as monomeric, trimeric, and multimeric forms. SPD multimer was the most prevalent, while the majority of SPD-MAA eluted as trimer and monomer. SPD dose-dependently bound to GFP-SP, but SPD-MAA binding to bacteria was significantly reduced. SPD enhanced, but MAA adduction of SPD prevented, both aggregation and macrophage phagocytosis of GFP-SP. Likewise, SPD increased bacterial permeability while SPD-MAA did not. In the presence of RSV, BEAS-2B cell viability was enhanced by SPD, but not protected by SPD-MAA. Our results demonstrate that MAA adduction changes the quaternary structure of SPD from multimer to trimer and monomer leading to a decrease in the native anti-microbial function of SPD. These findings suggest one mechanism for increased pneumonia observed in alcohol use disorders.

## Introduction

Alcohol misuse causes injury to the respiratory system as well as impeding lung repair and normal immune function (1). Alcohol use desensitizes ciliated upper airway epithelium resulting in diminished clearance of inhaled pathogens (2). In the lower airways, alcohol abuse causes failure to phagocytose and clear pathogens, induction of inflammatory cytokine release, upregulation and recruitment of inflammatory T cells, and up-regulation of pro-inflammatory transcription factors and pathways (1). Chronic alcohol use is associated with elevated cytokine levels resulting from increased oxidative injury in the lungs (3). Such oxidative inflammatory injury also results in the generation of reactive aldehydes such as malondialdehyde (MDA) (4). Acetaldehyde is another reactive aldehyde found in the lungs, not only in response to alcohol metabolism, but also because of smoking, where more than 0.5 mg/cigarette is inhaled (5). Cigarette and alcohol polysubstance use can adversely impact the respiratory system by depleting lung antioxidant levels, leading to chronic inflammation and increased susceptibility to bacterial infections (6, 7).

To minimize infection injury, the lungs have several innate defense mechanisms including the anti-microbial collectin, surfactant protein D (SPD). Secreted by alveolar type II cells and Club cells, SPD is a Ca^+2^ dependent lectin, preferring to bind to simple carbohydrates like glucose, mannose, and inositol (8, 9). SPD aggregates bacteria to enhance mucociliary transport in the upper airway and mediates macrophage phagocytosis of pathogens in the lower airways (8). In an environment not exposed to cigarette smoke or alcohol consumption, SPD is known to exist in several structures with varying functions. While monomers of SPD appear to have little function, large multimeric forms of SPD (such as dodecamers and even higher order structures) are strongly antimicrobial (10). Conversely, the trimeric form of SPD shows little antimicrobial characteristics and can even be pro-inflammatory (9).

We previously observed that the lungs of individuals who smoked cigarettes and were heavy alcohol drinkers formed MAA-adducted protein in response to the elevated amounts of lung acetaldehyde and malondialdehyde (11). Furthermore, we identified SPD to be one of these MAA-adducted proteins in mice (12). *In vitro* experiments demonstrated an adverse effect of SPD-MAA on lung epithelial cells and macrophages (7, 13). Therefore, we hypothesized that MAA adduction changes the structure of SPD from multimer to trimer/monomer and, in doing so, reduces the anti-microbial characteristics of SPD. Such a decrease in this important innate defensin through the covalent modification by reactive aldehydes would represent one of the injury mechanisms caused by alcohol and cigarette smoke to help explain the pathogenesis of increased pneumonia observed in alcohol misuse.

## Methods

### Purification and MAA-Adduction of Surfactant Protein D (SPD)

SPD was purified and adducted as previously described (7). Lung SPD was purified from human pulmonary alveolar proteinosis fluid (14). SPD was MAA-adducted by incubating 1-2 mg of SPD with a 2:1 ratio solution of malondialdehyde and acetaldehyde (SigmaAldrich, St. Louis, MO) for 3 d at 37°C in sealed polypropylene tubes as reported (15). Approximately 1–1.5 mg/mL of SPD was incubated with 1.0 mM acetaldehyde and 2.0 mM MDA in pyrogen-free 20 mM Tris buffer pH 7.4 containing 10 mM EDTA, 2 mM diethylenetriaminepentaacetic acid, and 2 mM Phytic acid in a sealed polypropylene vessel. The reaction was performed under sterile and non-oxidizing conditions in the dark for 72 h. At the end of incubation, the reaction mixture was exhaustively dialyzed under aseptic conditions against pyrogen-free phosphate buffered saline solution for 24 h at 4°C. As a handling control, mock-treated SPD was treated in the same manner in the absence of aldehydes.

The fluorescent 2:1 adduct formed during MAA adduct generation was quantified using a luminescence spectrophotometer excitation at 398 nm and emission maximum at 460 nm (Perkin Elmer, Norwalk, CT) and expressed as nanomole of fluorescent MAA equivalents per milligram protein.

### Gel Filtration Chromatography

Purified SPD and SPD-MAA were filtered using size-exclusion chromatography. An FPLC column (1.5 x 12 cm) (SigmaAldrich) was packed with 20 mL of Sephadex G-200 (Pharmacia Fine Chemicals, New York, NY) and equilibrated with 0.05 M HNaPO_4_ and 0.15 M NaCl (pH 7, 0.45 μm filtered). Purified SPD and SPD-MAA (1.5 mg/mL) were loaded onto separate columns and eluted into 12 x 1 mL fractions under isocratic conditions. Protein concentration of each fraction of SPD and SPD-MAA was quantified using a Bradford assay (16). Aliquots (10 μL) of each fraction and bovine serum albumin (BSA) standards (0, 0.125, 0.25, 0.5, 1, and 2 mg/mL) were diluted in 500 μL of Coomassie Blue (Bio-Rad, Hercules, CA) and absorption measured at 595 nm by visible spectrophotometry (Bio-Tek, Winsooki, VT).

### Western Blot

Eluted fractions were immobilized by slot blot (Bio-Rad Bio-Dot SF, Hercules, CA). Bio-Dot SF filter paper (Bio-Rad) and 1 sheet of 0.2 μm nitrocellulose membrane (Bio-Rad) were soaked in Western blocking buffer (0.05 M Tris, 0.15 M NaCl, pH 7.5) for 10 min. Samples were diluted 1:1000 in Western blocking buffer and 200 μL loaded into each slot. Nitrocellulose membranes were incubated in Western blocking buffer with 3% BSA (MilliporeSigma, Allentown, PA) overnight at 4°C. After a brief rinse in Western blocking buffer, primary antibody solution was added to the blot and rocked for 1 h at room temperature. Primary antibodies of goat-anti SPD (R&D Systems, Minneapolis, MN) and rabbit anti-MAA (17) were diluted 1:10,000 in Western blot buffer with 3% BSA. Blots were rinsed with blocking buffer for 20 min at room temperature with rocking, rinsed again with blocking buffer containing 0.02% NP-40 (SigmaAldrich) for 20 min with rocking, and lastly repeat washing with blocking buffer. Secondary antibodies for SPD (HRP-conjugated rabbit-anti Goat; Invitrogen, Carlsbad, CA) and SPD-MAA (HRP-goat anti-rabbit; Rockland, Limerick, PA) were diluted 1:15,000 in Western blocking buffer with 3% BSA and incubated for 1 h at room temperature. After repeat rinsing as described for primary antibodies, blots were incubated with ECL Western Blotting Substrate and Developer (ThermoFisher). Blots were exposed to X-ray film (PDC Healthcare, Valencia, CA) for 30 sec within 10 min after adding the developer to the nitrocellulose.

### Bacterial Preparation

*Streptococcus pneumoniae* expressing green fluorescent protein (GFP-SP) was a generous gift from the lab of Jan-Willem Veening in the Netherlands. *S. pneumoniae* were grown in Remel Mueller Hinton Broth with cations (calcium and magnesium), and laked horse blood (LHB) (ThermoFisher; Waltham, MA) until they reached log phase growth. The strep suspension was centrifuged at 1000 × *g* for 10 min and resuspended in 10% neutral buffered formalin for 10 min. The bacteria were subsequently washed three times in phosphate-buffered saline (PBS) and resuspended in PBS for further use.

### GFP-SP-SPD Binding Assay

SPD and SPD-MAA were biotinylated using a previously described method (18). Biotinylation of proteins was carried out by incubating with Immunopure NHS-LC-Biotin (Pierce, Rockford, IL) at a ratio of 2:1 of biotin to collectin by weight for 2 h at room temperature in the dark. Unbound biotin was removed by overnight dialysis. SPD and SPD-MAA binding to bacteria was tested by ELISA where *S. pneumoniae* (1 μg/ml) was dried onto 96-well plates (Falcon, Glendale, AZ) and fixed with methanol (MilliporeSigma) as described (19). Non-specific binding was blocked using BSA (MilliporeSigma) and gelatin (Bio-Rad) before incubation with biotinylated SPD. MAA-adducted BSA (BSA-MAA) was used as a control against any non-specific MAA adduct artifact. Bound biotinylated protein was detected with streptavidin conjugated to horseradish peroxidase followed by TMB substrate (Bio-Rad). Reactions were halted with 1 N H_2_SO_4_ and optical density measured with an ELISA plate reader using visible photospectroscopy (Bio-Tek).

### Cell Culture

BEAS-2B bronchial epithelial cells and Raw 264.7 macrophages were purchased from American Type Cell Culture (ATCC, Rockville, MD) and cultured in Dulbecco’s modified Eagle’s medium (DMEM) (Gibco, Grand Island, NY) supplemented with 10% fetal bovine serum (FBS, Atlantis Biosciences, Singapore) and 1% penicillin/streptomycin (Gibco) and maintained at 37°C in a humidified CO_2_ incubator (Panasonic, Wood Dale, IL).

### Aggregation Assay

The aggregation assay was performed as previously described (18, 20) in the presence or absence of 5 mM calcium. PBS was used to acquire a final volume of 50 μL. A Zeiss Axio inverted fluorescence microscope (Zeiss, Oberkochen, Germany) was used to visualize aggregation. An average of 10 fields of view were examined per slide. Suspensions (50 µl) of GFP-SP were incubated with SPD (10 µg/ml) for 90 min at 37°C in the presence or absence of 5 mM CaCl_2_. Samples were placed on slides and examined by phase-contrast and fluorescence microscopy (magnification, 50X). Ten fields of view were counted per slide, and the average area and number of clumps of aggregated bacteria per field of view was determined.

### Phagocytosis Assay

Phagocytosis of GFP-SP was performed as previously described (21). RAW 264.7 cells were incubated overnight in media onto coverslips in a 12-well plate. RAW cells were then incubated for 30 min in a final 500 µL suspension of SPD or MAA-SPD in PBS with or without 5 mM calcium. Cells were then incubated with 100 µL of bacterial suspension for 60 min, quenched with 1 mL Trypan Blue (Gibco)/well for 3 min, washed 2X with PBS, fixed with 0.5% formalin (ThermoFisher) for 10 min, and then washed again. Coverslips were mounted onto slides and fluorescence was visualized by fluorescence microscopy.

### Permeability Assay

The permeabilizing effects of SPD and SPD-MAA on *S. pneumoniae* were assayed as a function of bacterial phosphatase release using a commercial endogenous phosphatase detector kit (Thermo Fisher).

### Anti-Viral Protection Assay

BEAS-2B (2 x 10^5^ cells/mL) were cultured in 24-well tissue culture plates for 2 d, until approximately 85% confluent. Respiratory syncytial virus (RSV-2A; Advanced Biotechnologies; Eldersburg, MD) was diluted to 0.1 MOI in DMEM without serum or antibiotics in the presence of 0, 10, and 100 µg/mL SPD or SPD-MAA in a total volume of 0.5 mL and incubated for 1 h at 4°C. Cells were washed in PBS to remove serum and antibiotics and 200 µL per well of each RSV treatment condition was added to a well for 2 h at 37°C. After 2 h, an additional 200 µL of DMEM with penicillin/streptomycin was added (post-inoculation) and cells incubated for 48 h at 37°C. Cell supernates were then collected, dead cells pelleted at 200 g for 10 min, and media decanted. Pelleted cells and remaining cells attached in wells were assayed for total protein by Bradford. As previously reported for RSV-infected BEAS-2B (22), lactate dehydrogenase (LDH) activity was measured in the supernatant media using the LDH Activity Assay Kit (Sigma-Aldrich) according to the manufacturer’s instructions. Cell homogenates were added to the LDH assay buffer and LDH substrate mix. Absorbances were measured at 595 nm by visible spectrophotometry (Bio-Tek). Each sample was standardized by total protein.

### Statistical Analysis

All experiments were performed a minimum of 5 times (n=5). Each data point graphically presented represents the standard deviation of those experiments. Data were analyzed using Graph Pad Prism (v9.2.0 for Mac, GraphPad Software, San Diego CA). Data were analyzed for statistical significance using a non-parametric Kruskal-Wallis test. Significance was accepted at the 95% confidence interval.

## Results

### MAA Adduction Changes the Structure of SPD

**Figure 1:**
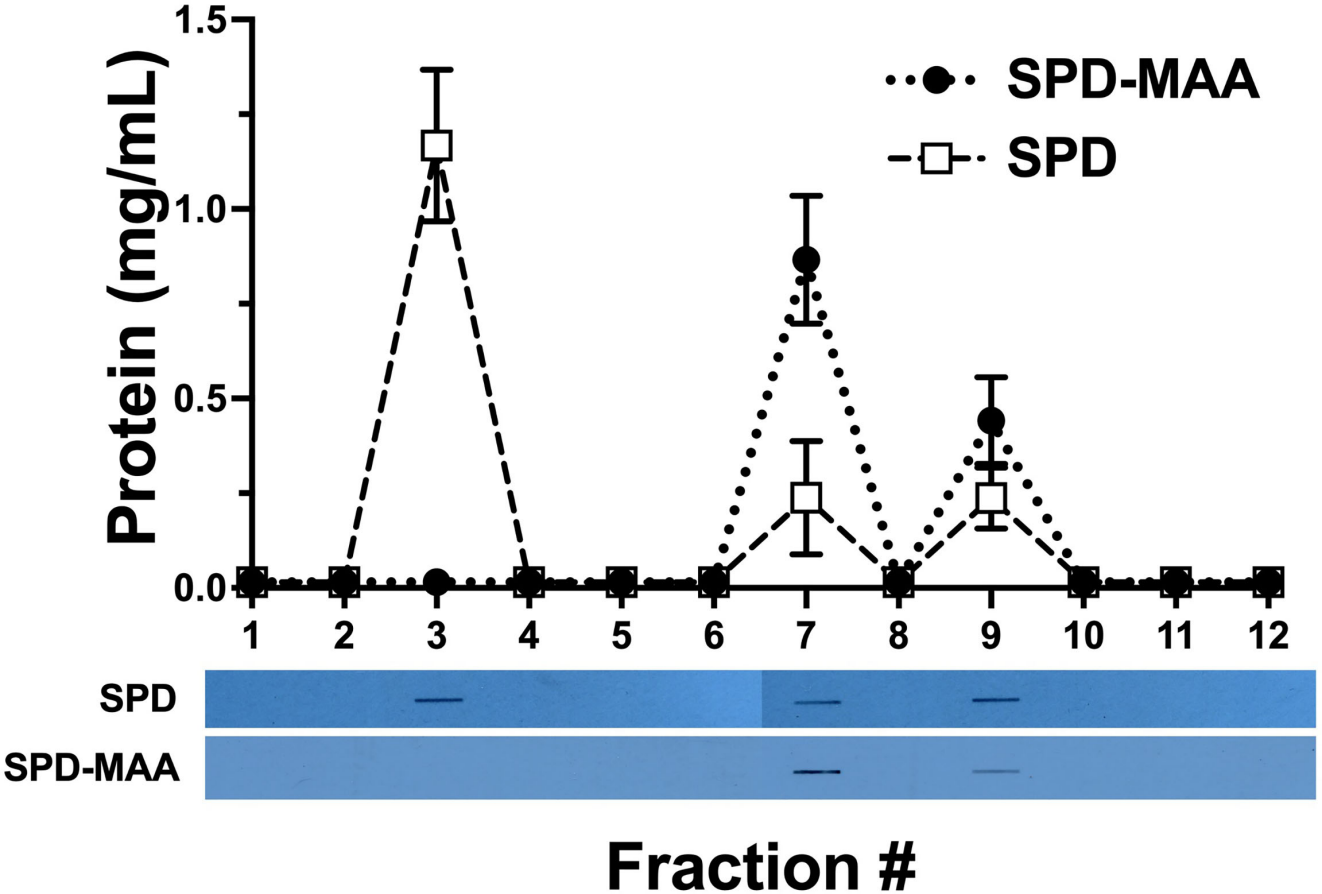
Bradford protein assay with corresponding bands from the Western blot. Column fraction protein concentrations were quantified using a Bradford assay. Protein was detected in 3 fractions eluted by gel filtration. In SPD, most of the protein (multimer) was collected in Fraction 3. In SPD-MAA, almost no multimer eluted while trimer (Fraction 7) and monomer (Fraction 9) were collected. Western blots for each slot blotted fraction were probed for SPD and SPD-MAA. The SPD column fractions showed the presence of SPD in 3 fractions. SPD-MAA column fractions only contained SPD in Fractions 7 and 9. Blots probed with anti-MAA detected protein from SPD-MAA column fractions 7 and 9 (not shown).

Purified SPD and SPD-MAA (1.5 mg/mL) were each resolved by size exclusion chromatography and measurable protein detected by Bradford assay in eluted fractions 3, 7, and 9 (**Figure 1**). Three fractions corresponding to multimer (>589 kDa), trimer (55 kDa), and monomer (18 kDa) were collected from SPD with multimeric protein eluting out of the column first in fraction 3. Only two SPD-MAA fractions containing protein were eluted as fraction 7 and 9, corresponding to trimer and monomer. Western blot using goat anti-SPD revealed the presence of SPD protein in all 3 fractions for purified SPD, but only fractions 7 and 9 for purified SPD-MAA. Western blot using rabbit anti-MAA detected MAA-adducted protein in fractions 7 and 9 for SPD-MAA. No reactivity for anti-MAA was observed in the fractions eluted from non-adducted, purified SPD. As expected, these data confirm that purified non-adducted SPD predominately exists as multimer, trimer, and monomer, but most of the SPD-MAA elutes as trimer and monomer with no detection of the multimeric form.

### MAA Adduction Decreases SPD Binding to Bacteria

**Figure 2:**
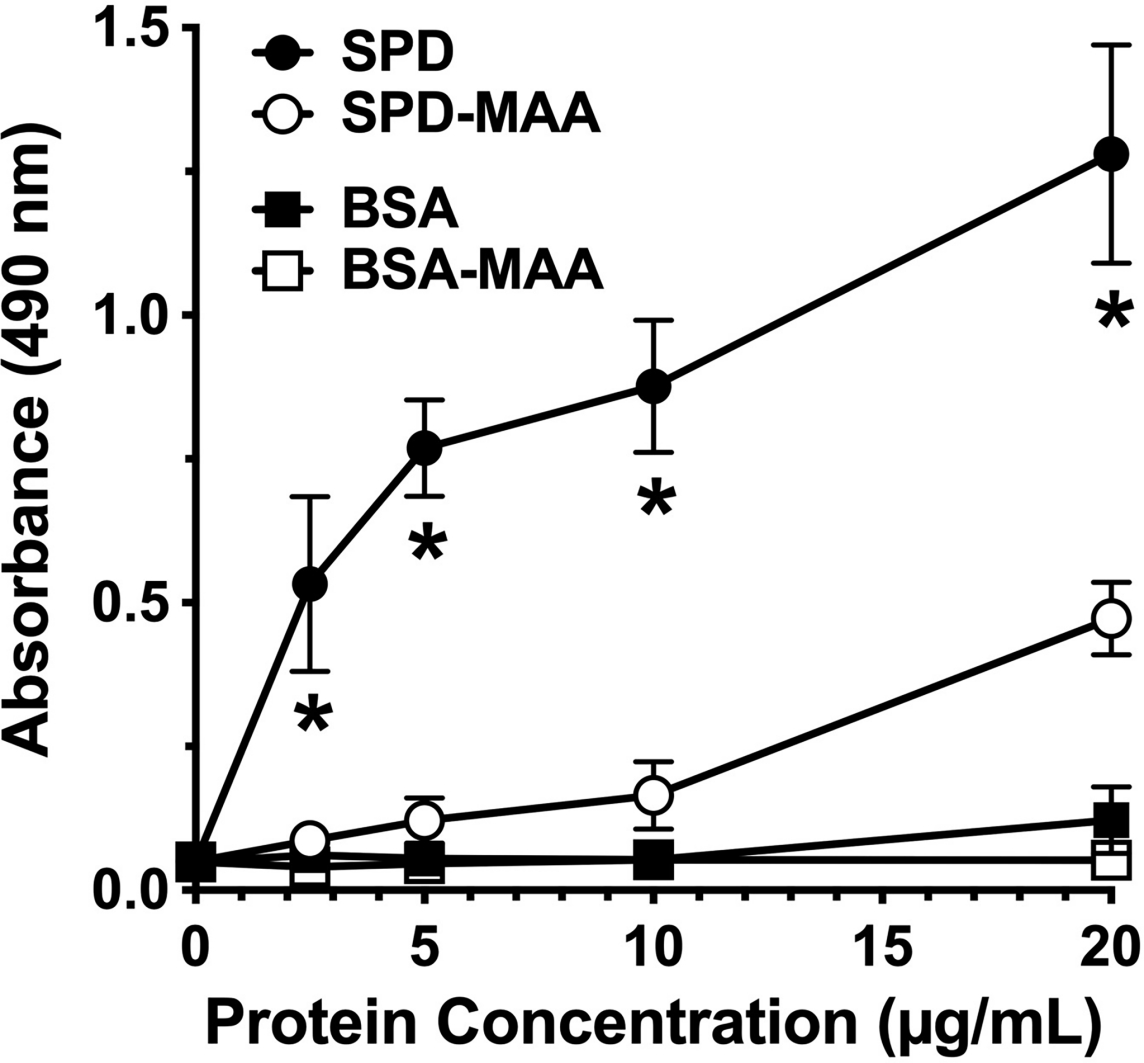
Surfactant protein binding to *S. pneumoniae*. Plates were coated with bacteria and incubated with 0-20 µg/mL SPD, SPD-MAA, BSA (negative control), or BSA-MAA. Bound SPD was detected with antibodies by ELISA. Absorbance at 450 nm of bound SPD at the different concentrations was determined (n = 5), non-parametric Kruskal-Wallis test was performed, differences between SPD and SPD-MAA were significant at all concentrations (*p< 0.01). The data are the averages +/- SD of five experiments. The only significant difference between SPD-MAA and the BSA negative control was observed at 20 µg/mL. No binding was observed for BSA-MAA.

SPD exhibits loss of bacterial binding in trimeric form as compared to the native multimeric form [Arroyo 2020]. To determine whether SPD-MAA exhibits reduced bacterial binding compared to SPD, we conducted binding assays using *S. pneumoniae* and biotinylated surfactant proteins. SPD bound to bacteria in a dose-dependent manner with maximum binding between 10-20 µg/mL SPD (**Figure 2**). In contrast, SPD-MAA showed no significant binding to *S. pneumoniae* except at the highest concentration (20 µg/mL) tested. At this concentration, SPD bound significantly higher (p<0.01) than SPD-MAA. No bacterial binding was detected with BSA or BSA-MAA. These data reveal a functional difference in SPD bacterial binding when the protein is MAA-adducted.

### MAA Adduction Decreases SPD Aggregation of Bacteria

**Figure 3:**
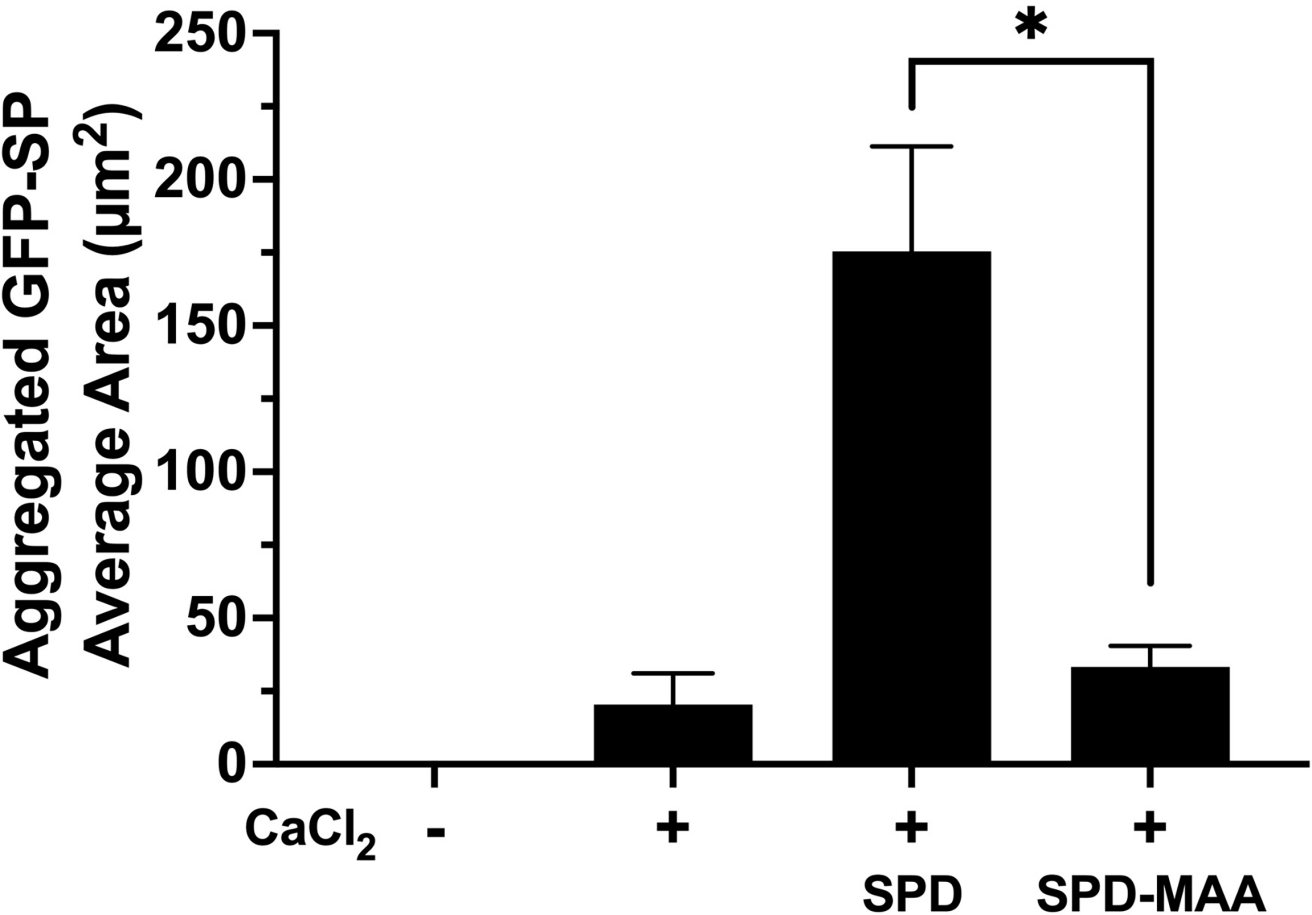
Aggregation of *S. pneumoniae* in the presence of surfactant protein. GFP-SP were incubated with 10 µg/ml SPD or SPD-MAA for 90 min in the presence of calcium (5 mM) and visualized by fluorescence microscopy (X 500). The average area of aggregated clumps of bacteria was measured per field of view. The data are the averages +/- SD of five experiments. In the presence of calcium, SPD significantly increased aggregation compared to SPD-MAA, *p<0.0003. No clumping or aggregation of pneumococci was observed in the absence of calcium.

As the term collectin suggests, SPD aggregates bacteria to enhance opsonization and phagocytosis. Using GFP-SP in the absence of any cells, we observed a diffuse punctate dispersal of bacteria by fluorescence microscopy (**Figure 3**). Upon the addition of 5 mM calcium, *S. pneumonia* can form small clumps in culture. In the presence of 10 µg/mL SPD, large aggregates of GFP-SP were readily evident. However, at the same concentration, SPD-MAA failed to aggregate bacteria and produced no additional clumping beyond that observed with calcium alone. These data identify a functional difference in bacterial aggregation between SPD and SPD-MAA that is consistent with the reported difference between SPD multimer vs. trimer forms.

### MAA Adduction Decreases SPD Enhancement of Phagocytosis

**Figure 4:**
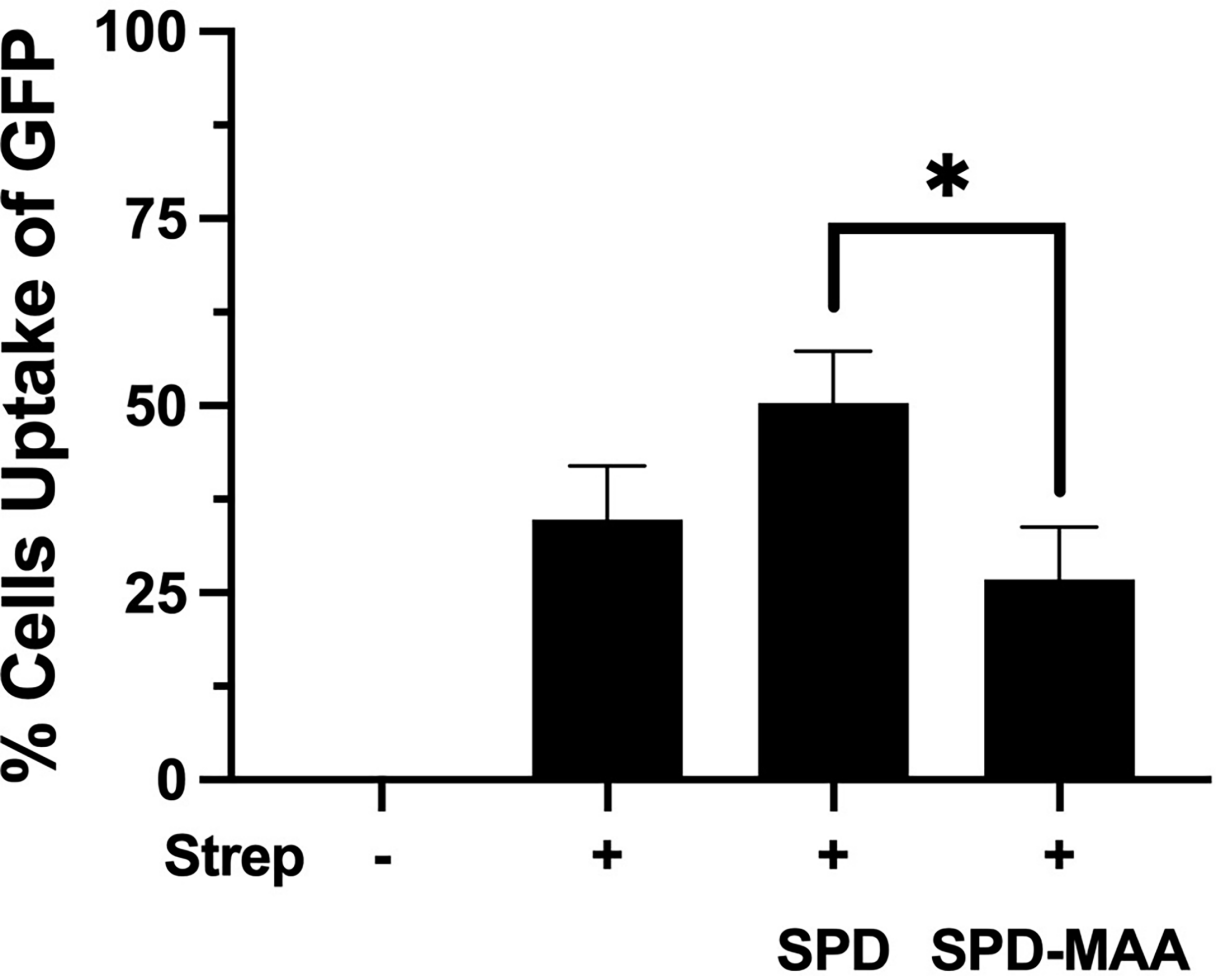
MAA adduction of SPD decreases phagocytosis of *S. pneumoniae*. GFP-*S. pneumoniae* were incubated with the murine macrophage cell line RAW264.7. The percent of RAW264.7 cells containing internalized bacterial cells per field was determined using fluorescence microscopy (X 500) after quenching non-internalized GFP-SP with Trypan blue. The image results are a representative of 5 independent experiments summarized by graph. Bars represent averages +/- SD, *p<0.0004.

Phagocytosis of *S. pneumoniae* is enhanced by the binding and aggregation of SPD to bacteria. We evaluated the impact of MAA adduction on the SPD-mediated *S. pneumoniae* phagocytosis by macrophages in an *in vitro* assay. RAW 264.7 macrophages were incubated with GFP-SP in the presence or absence of 10 µg/mL SPD or SPD-MAA. Cultures were then washed, and Trypan blue was used to quench non-internalized GFP-SP fluorescence allowing for visualization of only internalized GFP-SP by fluorescence microscopy. GFP-SP was phagocytosed by 25-50% of the RAW 264.7 cells per each field of view (**Figure 4**). The addition of 10 µg/mL SPD enhanced the average number of cells phagocytosing GFP-SP. However, SPD-MAA had no significant enhancement effect on bacterial uptake by the macrophages. A significantly different level of phagocytosis was observed between SPD and SPD-MAA (p<0.0004). These data suggest that MAA adduction of SPD prevents surfactant protein enhancement of phagocytosis.

### MAA Adduction Decreases SPD Enhancement of Bacterial Permeability

**Figure 5:**
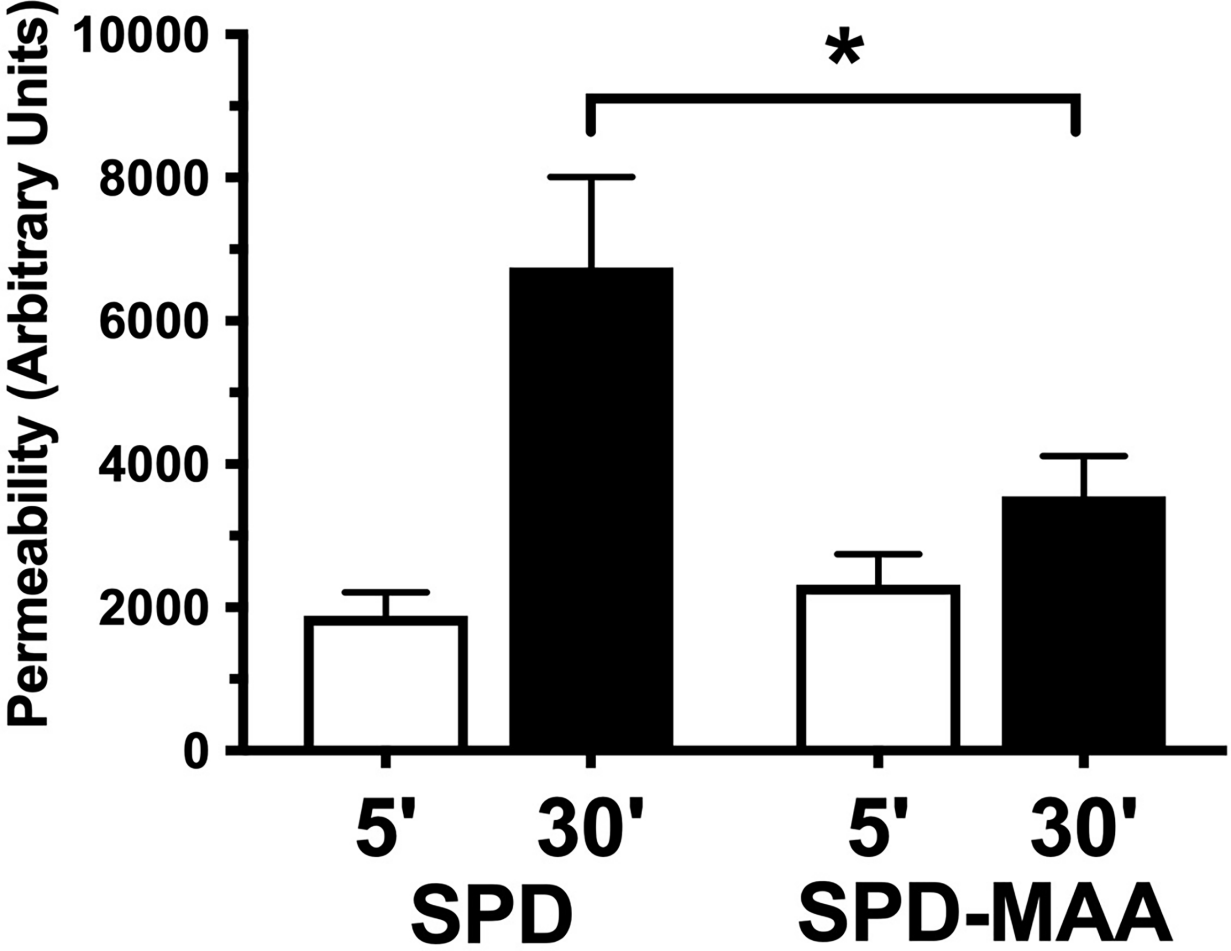
SPD-induced anti-bacterial permeability is decreased by MAA adduction. *S. pneumoniae* was incubated in the presence of either 10 µg/mL SPD or SPD-MAA for up to 30 min and supernatant media phosphatase release measured as a function of bacterial permeability. A significant reduction in permeability (*p<0.05) was observed between SPD and SPD-MAA. Bars represent averages +/- SD, n=5 experiments.

Surfactant can engage in direct anti-microbial action through increasing bacterial membrane permeability. To determine the effect of MAA adduction on SPD anti-bacterial killing, we assayed membrane permeability as a function of endogenous phosphatase release in cell-free *in vitro* suspensions of *S. pneumoniae* and surfactant. After 30 min of incubation with 10 µg/mL SPD, we detected a significant increase (p<0.01) in endogenous phosphate release vs. no SPD (**Figure 5**). However, the ability of SPD-MAA to permeabilize bacteria was significantly (p<0.05) decreased compared to that of SPD. These data support that MAA adduction reduces anti-bacterial killing.

### MAA Adduction Decreases SPD Anti-Viral Protection

**Figure 6:**
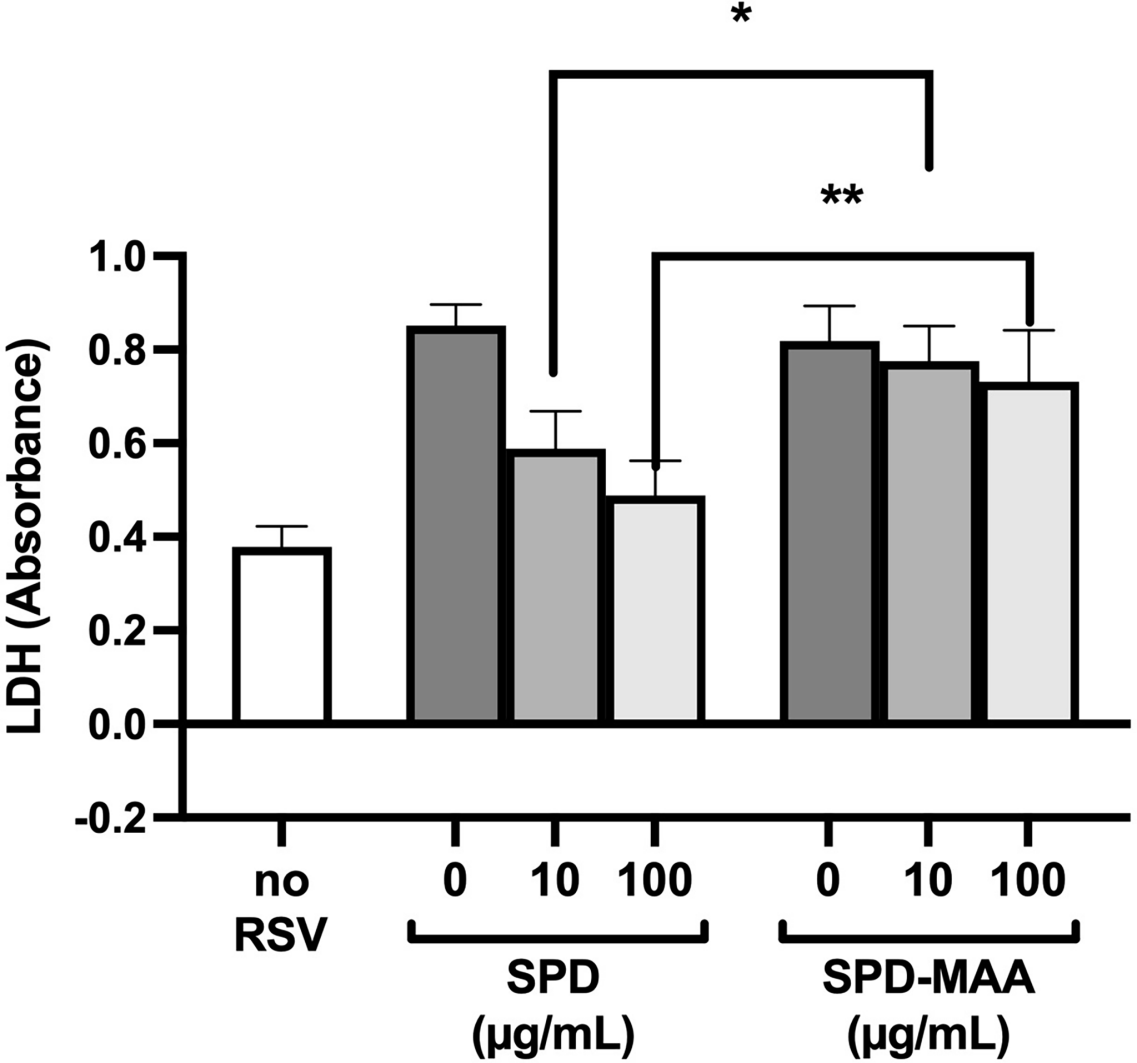
MAA adduction of SPD decreases cell viability in response to respiratory syncytial virus (RSV). Infection of BEAS-2B with RSV increases LDH release compared to control media (no RSV). SPD (10-100 µg/mL) significantly (p<0.0002) decreases RSV-induced LDH release vs. the absence of SPD. No such decrease is observed with either 10 µg/mL (*p<0.01) or 100 µg/mL (**p<0.006) SPD-MAA vs. equal amounts of SPD. Bars represent averages +/- SD, n=5 experiments.

The anti-microbial innate defense by SPD extends to anti-viral protection. RSV is a pathogen that specifically infects the airway epithelium resulting in cell detachment and death. To determine if surfactant protein protection from RSV is impacted by MAA adduction, we infected bronchial epithelial BEAS-2B cells with RSV in the presence or absence of 10-100 µg/mL SPD or SPD-MAA and measured LDH as a marker for cell viability. RSV caused a decrease in cell viability, but the addition of 10 or 100 µg/mL SPD significantly (p<0.0002) decreased the amount of LDH detected (**Figure 6**). In contrast, SPD-MAA produced no protection from cell death, as increased LDH remained after treatment with 10 µg/mL (p<0.01) and 100 µg/mL (p<0.006) SPD.

## Discussion

Chronic alcohol use predisposes an individual to a plethora of maladies including chronic liver disease, chronic kidney disease, cancer, and respiratory disease (23). Alcohol is particularly important in pulmonology as alcohol misuse is associated with increased risk of acquiring pneumonia, developing antibiotic resistant pneumonia, and pneumonia severity compared to individuals who do not abuse alcohol (24). Alcohol abuse has been shown to leave an individual more at risk of contracting respiratory illness from *S. pneumonia, L. pneumophila*, and gram-negative enteric bacilli (24).

In 2019, an estimated 14.5 million Americans over the age of 12 were impacted by alcohol use disorder (AUD) (25). Already classified as one of the top 3 lifestyle-related causes of death in the United States (26), alcohol misuse is expected to rise due to COVID-19 (27), thus underscoring the importance of understanding alcohol effects on tissue injury.

As with alcohol, cigarette smoking is the leading cause of preventable death and disease in America (28). Cigarette smoking is an established cause of several health ailments, including impairing the respiratory system’s ability to function, protect, and repair itself (29). With 34.1 million active Americans adult smokers in 2019, the burden that cigarettes place on individual and public health results in nearly $300 billion dollars of smoking-related costs each year (28). Cigarette smokers incur up to a fourfold increased risk ratio of developing pneumonia than non-smokers (29). Cigarette smokers have increased white blood cell counts, specifically with regard to CD4^+^ and CD8^+^ lymphocytes, cytokines (IL-1, IL-8, TNF alpha, and granulocyte-macrophage colony-stimulating factor), and decreased phagocyte activity (29).

The combined use of alcohol and cigarettes introduces a new set of potential risk factors to respiratory disease and immune function. Most individuals with an AUD also use cigarettes (6). Evidence suggests that individuals consuming nicotine are more likely to overconsume alcohol (30). It is known that co-exposure to cigarette smoke and alcohol use is uniquely negative to the respiratory system. The proposed mechanism for this damage is through reactive aldehydes generated during the metabolism of both cigarette smoke and alcohol (11). Cigarette smoke contains acetaldehyde (AA) and several reactive oxidative species (ROS), and when inhaled cause oxidative stress (31). ROS can cause lipid peroxidation in the liver leading to the synthesis of malondialdehyde (MDA) (15, 31). Alcohol metabolism similarly leads to the AA formation through CYP2E1 and alcohol dehydrogenase (12). CYP2E1 also generates ROS, leading to lipid peroxidation in the liver which forms MDA (12). ROS can lead to inflammation and cytokine release, activating neutrophils and monocytes (4). Chronic inflammation from this can lead to DNA damage, cancer, and inhibition of apoptosis, leading to illnesses like chronic obstructive pulmonary disorder (COPD) (4). AA can form adducts to proteins and DNA, which can lead to robust inflammatory responses (12). AA and MDA can react through a Schiff base intermediate to generate hybrid malondialdehyde-acetaldehyde adducts, or MAA adducts (32). MAA adducts are stable and can covalently bond to proteins at lysine residues (15, 33).

One of the first lines of defense the body has is innate defense mechanisms; in the respiratory system this includes SPD. The simplest unit of SPD is the monomer, which lacks discernable function on its own (10). SPD monomer structure consists of 355 amino acids (43 kDa) arranged into 4 subunits: a short N-terminal domain, a long collagen region, alpha-helical coiled neck domain, and a C-terminus with a carbohydrate recognizing domain (8, 9). The neck and head region of SPD are stabilized by 2 Ca^+2^ ions and 2 disulfide bonds (9). The carbohydrate recognizing domain is located at amino acids Glu 321 and Asn 323, in order to bind to carbohydrates a glycoprotein is required (9).. The long collagen region is a repeating sequence of Gly-X-Y, a region thought to be responsible for oligomerization of SPD and interacting with scavenger receptor A (SRA). SRA is the hypothesized macrophage receptor that interacts with SPD to generate its immune functions (9). The short N-terminus is composed of 2 Cys located in aa15 and aa20 allowing for an interchain disulfide crosslinking to form which stabilized SPDs trimer structure (9). Three monomers can oligomerize to a trimer through the assembly of the collagen regions into triple helices and a coiled bundle made of alpha-helical neck regions (8). Trimers lack the protective immune functions of high order structures but can still bind to 2-3 glycoconjugates due to the spacing of the heads (9, 10). High level of trimerized SPD could inhibit higher order oligomer functions, such as bacterial aggregation and phagocytosis, but encourage inflammation (9). Two trimers can form a hexamer, the next higher order structure of SPD found *in vivo*. Hexamers are a structural intermediate, found as either V-shaped or rod-shaped forms, and bind and aggregate 50-60% of available bacteria (8, 10). Four trimers make up a dodecomer structure, which is the most abundant structure *in vivo* (8). Dodecamers and other higher order structures (fuzzy balls) have shown to be strongly antimicrobial (10). The formation and distribution of SPD structures was largely facilitated by the immediate environment’s pH (8). Dodecamers and other higher order oligomers are associated through the N-terminus of trimer subunits (9).

**Figure 7:**
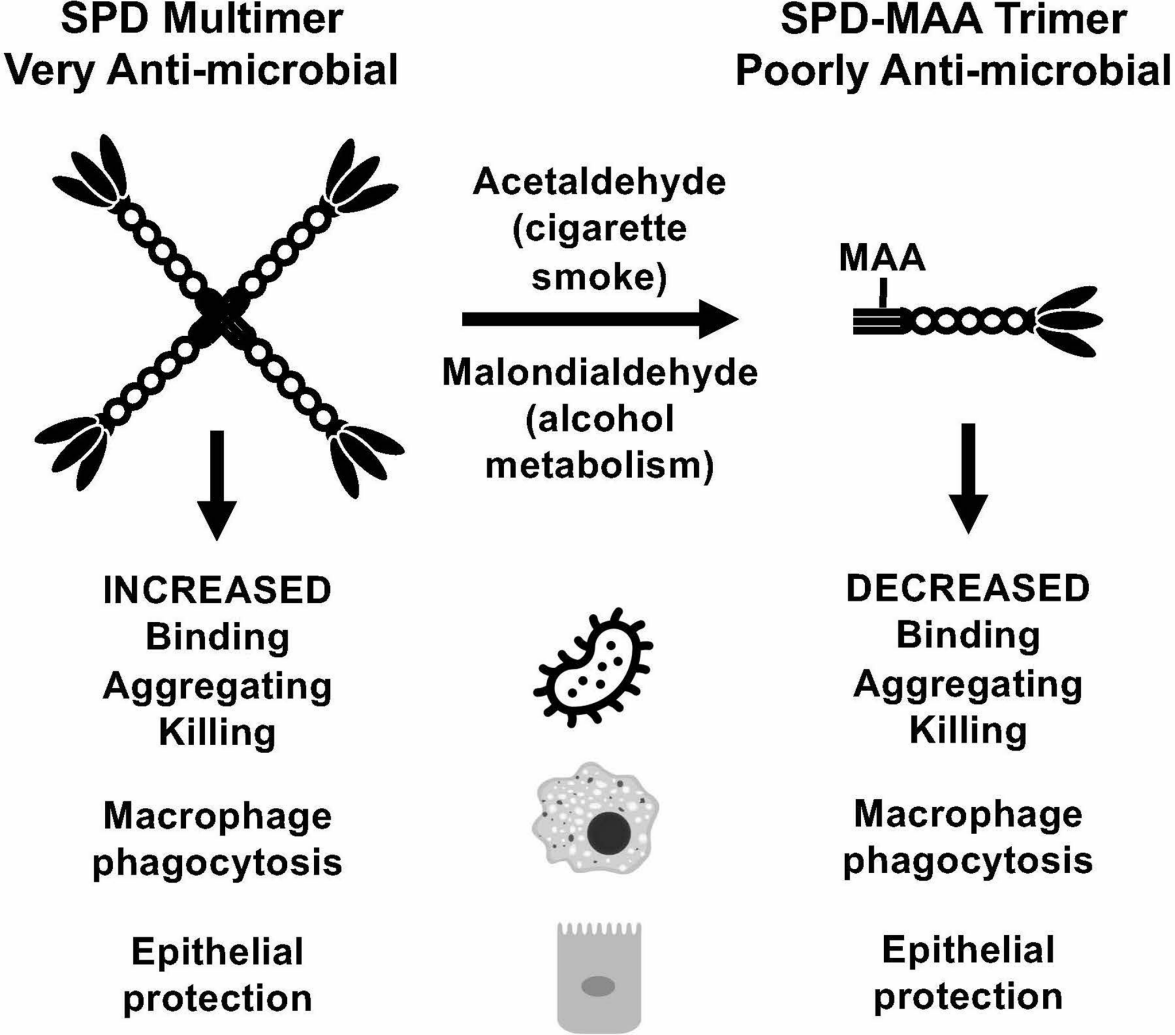
Summary model. Covalent modification of surfactant protein D (SPD) by malondialdehyde-acetaldehyde (MAA) adduction changes the structure of the protein from multimeric form to trimeric form and reduces anti-microbial innate defense.

Inactivation of SPD through N-terminus modifications can occur through several known modifications including nitrosylation of the cysteine-residues, which also results in a change in the structure from an oligomeric form to a trimeric form (34). While by no means the only means of SPD structural change, MAA adduction occurs primarily on lysine residues. At the N-terminus of SPD, which governs quaternary structure, is a lysine-containing target for possible adduction. It is the N-terminal region of each SPD subunit that is responsible for the formation of multimeric structures (9). When MAA adducts to SPD to form SPD-MAA, it forms a stable intermediate (**Figure 7**) that is not easily degraded and leads to altered immune effects (12). SPD-MAA adducted proteins bind to scavenger receptor A (CD204) before being internalized to trigger an inflammatory response (31). SRA is found on immune cells such as macrophages, epithelial cells, dendritic cells, and the endothelium (31). Ligand-bound SRA activates PKC_ϵ_ to recruit more neutrophils, keratinocyte chemoattractant (KC), and release inflammatory signal molecules and chemokines including: TNFα, IL-6, IL-8, IL-12 (12, 35, 36). Due to the decreased protective functions of SPD once it has been MAA adducted, we observed that SPD-MAA functionally resembles the SPD trimer structure that lacks the innate immune properties of higher order oligomers such as the dodecomer (**Figure 7**). Our size exclusion results confirmed that native SPD predominately exists in multimeric form while SPD-MAA exists as trimer and monomer structures. Our findings provide a potential mechanism for the previous study where a reactive aldehyde contained in cigarette smoke, acrolein, also resulted in decreased SPD function (37). This shift in SPD structure from multimer to trimer due to MAA adduction replicates the previously reported decreases in SPD binding, aggregation, and killing of bacteria (10). In addition, macrophage phagocytosis of *S. pneumoniae* was no longer enhanced when SPD was MAA adducted. As well as being antibacterial, SPD is an endogenously produced antiviral protein (9). We observed that bronchial epithelial cell death due to RSV infection was significantly reduced in the presence of SPD. This protection was lost when SPD was MAA adducted. Because SPD is the key surfactant in the lungs that binds to the S-protein of SARS-CoV-2 (38, 39), decreased SPD protection against COVID-19 may be similar to that of RSV. Loss of innate defense at the level of SPD in alcohol misuse may explain one of the mechanisms for alcohol comorbidities observed in the COVID-19 pandemic (10, 40).

## Data Availability Statement

The raw data supporting the conclusions of this article will be made available by the authors, without undue reservation.

## Author Contributions

CN wrote the manuscript and conducted experiments. DM and KB conducted experiments and edited the manuscript. KK prepared SPD-MAA and edited the manuscript. TW designed, analyzed data, wrote, and edited the manuscript. All authors contributed to the article and approved the submitted version.

## Funding

Support was obtained from Central States Center for Agricultural Safety and Health (CS-CASH; U54 OH010162 to TW), VA Merit (I01 BX003635 to TW and I01 BX005413 to TW and KB), and National Institute on Aging (R01 AG0535553 to KB). TW is the recipient of a Research Career Scientist Award (IK6 BX003781) from the Department of Veterans Affairs.

## Conflict of Interest

The authors declare that the research was conducted in the absence of any commercial or financial relationships that could be construed as a potential conflict of interest.

## Publisher’s Note

All claims expressed in this article are solely those of the authors and do not necessarily represent those of their affiliated organizations, or those of the publisher, the editors and the reviewers. Any product that may be evaluated in this article, or claim that may be made by its manufacturer, is not guaranteed or endorsed by the publisher.
